# Alzheimer y su Diagnóstico: ¿Estamos Preparadas Para Acompañar a Nuestras Pacientes? La Planificación Compartida de la Atención Como Marco Relacional

**DOI:** 10.31083/RN42462

**Published:** 2025-05-26

**Authors:** Míriam Eimil-Ortiz, Pilar Alcántara-Miranda, Sonia Martínez-Morante

**Affiliations:** ^1^Servicio de Neurología, Hospital Universitario de Torrejón (HUT), 28850 Madrid, Spain; ^2^Miembro del Grupo de Planificación Compartida de la Atención del H.U.Torrejón, Madrid, Spain; ^3^Instituto de Neurociencias, Hospitales Vithas, Madrid, Spain; ^4^Instituto de Neurociencias Madrid, Hospitales Vithas, Madrid, Spain

Estamos viviendo una transformación del paradigma de la Enfermedad de Alzheimer. Se cuestiona la propia definición de “Enfermedad”, planteándose la posibilidad de sustituir la clínica por la biología [1, 2]. Las especialistas que confiamos en un proceso diagnóstico que parte de síntomas y signos para diseñar con el paciente el mejor manejo posible desde la primera consulta nos encontramos ante un cambio radical que va más allá de un problema de nomenclatura [3].

Aunque en este punto podríamos ya plantear muchos elementos para la reflexión [científicos y éticos], nos queremos centrar en algo que, como facultativas clínicas a pie de calle y con amplia experiencia en el seguimiento de enfermos y familiares, nos preocupa mucho: ¿Cómo abordaremos en consulta el acompañamiento de personas diagnosticadas de Enfermedad de Alzheimer? Enfatizamos que estamos en un momento en el que no existe tratamiento que haya demostrado sin fisuras actuar sobre el trasunto biológico de la enfermedad y en el que ya hay herramientas disponibles en el mercado que pueden “sentenciar de enfermedad”, con un amplio margen de precisión [4], a la persona de una enfermedad neurodegenerativa, progresiva e incurable.

Si bien esta cuestión no es nueva, insistimos en llevar a primera línea algo fundamental: el paciente ha de estar en el centro de su atención, desde el mismo momento en que se plantea la posibilidad de este diagnóstico. Debemos preparar a los pacientes para recibir determinados resultados antes de solicitarles más estudios [5], repasar con ellos el motivo de consulta [6] y advertirles de las posibles incertidumbres a pesar de una exhaustiva investigación [7]. El diagnóstico precoz de la EA es beneficioso para el paciente y su entorno [8], pero debemos saber hacerlo [9] y, lo más importante, encuadrarlo dentro de una estrategia mucho más global: La Planificación Compartida de la Atención (PCA).

Y aquí es adonde nos interesa llegar. La PCA es “un proceso comunicativo-deliberativo, relacional y estructurado, que facilita la reflexión y la comprensión de la vivencia de la enfermedad y el cuidado, centrado en la persona, de quienes afrontan una trayectoria de enfermedad, para identificar y expresar sus valores, preferencias y expectativas de atención. Su objetivo es promover la toma de decisiones compartida en relación con el contexto actual y los retos futuros de atención, como en aquellos momentos en que la persona no sea competente para decidir” La descripción del proceso de Planificación Compartida no es sencilla en unas líneas, para ello remitimos al artículo de Pérez de Lucas y col [10] donde se explica el proceso: a lo largo de conversaciones sostenidas en consulta y por medio de técnicas como el counselling se: (1) explora conocimiento del paciente sobre su enfermedad, y, además, su capacidad de decidir (2) analiza su historia de vida, con sus valores primordiales, sus vivencias (3) averigua su sentir sobre la enfermedad (4) planifica respecto de situaciones presentes y futuras y se define un representante para casos de incapacidad (5) se documenta en la historia clínica y (6), siempre, siempre, se revisa cíclicamente aprovechando momentos de cambio.

La PCA va mucho más allá de la cumplimentación de unas instrucciones previas o de la mera exposición de pros y contras de una terapia o de un proceso diagnóstico. Es un complejo proceso en el que, por medio de una escucha activa, se identifica con el paciente y su entorno su conocimiento sobre su enfermedad, se explora su respuesta emocional ante ella y se averiguan sus valores, en el contexto de una historia de vida, donde está la persona y quienes le acompañan. Sirve para tomar decisiones de un modo compartido para situaciones actuales y futuras y se documenta en la historia clínica. Y esto se revisa periódicamente, porque las situaciones cambian y las necesidades se modifican con el curso de la enfermedad. Una parte fundamental de este proceso, y mucho más en el ámbito de la demencia, es la elección de una persona representante, cuya participación en las conversaciones irá siendo cada vez más importante, pues no podemos obviar que nuestras personas con demencia irán perdiendo facultades cognitivas y aquel irá asumiendo progresivamente un papel cada vez más protagonista. Pero ese protagonismo siempre será delegado, esto es, no podremos olvidar que la toma de decisiones, independientemente de lo avanzado de la enfermedad, deberá tener en cuenta los valores que la persona enferma una vez nos contó en consulta.

Creemos, sí, que un diagnóstico precoz permite que el paciente esté mejor preparado para la enfermedad. Pero también creemos que esa persona precozmente diagnosticada necesitará acompañamiento a lo largo de toda su enfermedad, incluyendo las etapas finales, y que todo este seguimiento se puede hacer en el marco de la PCA.

Sostenemos que es imprescindible la incorporación de la PCA para acompañar a la triada constituida por la persona con demencia/representante y su entorno. Ni nuestras limitaciones ni las condiciones cognitivas de nuestros pacientes deben ser empleadas como excusa para no trabajar en esa dirección. Poner en práctica esto requiere de formación y también de cooperación entre niveles. No olvidemos que nuestras personas con demencia se mueven en un entorno constituido por su familia, su comunidad y son asistidas, en primer lugar, por su médico de familia. Desde la convicción de esta necesidad seremos capaces de mover a las instituciones para que nos lo faciliten y de promover entre todos un cambio de mirada para que los profesionales que atendemos a las personas enfermas y sus representantes podamos ejercer la Neurología que queremos.

Sólo desde esta perspectiva humanizadora de la relación podremos asegurar que estamos preparadas para iniciar el diagnóstico precoz de la enfermedad de Alzheimer y acompañarla en todo su recorrido.

## Contribuciones de los Autores

MEO wrote the first draft, PAM and SMM made substantial contributions to the first version. All authors contributed to editorial changes in the manuscript. All authors read and approved the final manuscript. All authors have participated sufficiently in the work and agreed to be accountable for all aspects of the work.

## Aprobación Ética y Consentimiento Informado

No aplicable.

## Agradecimientos

No aplicable.

## Financiación

Esta investigación no recibió financiación externa.
